# Safety and effectiveness of ferric citrate hydrate in serum phosphorus management of patients with chronic kidney disease: a long-term, real-world, observational, post-marketing surveillance study

**DOI:** 10.1007/s10157-022-02204-1

**Published:** 2022-03-08

**Authors:** Keitaro Yokoyama, Teruo Hashimoto, Yuri Okuda, Yu Matsumoto, Kyoko Ito, Ryoichi Yamada, Hiroyuki Susai, Noriaki Nishino

**Affiliations:** 1 Department of Health Science, The Graduate School, The Jikei University School of Medicine, 3-25-8 Nishi-Shinbashi, Minato-ku, Tokyo, 105-8461 Japan; 2 Pharmaceutical Division, Japan Tobacco Inc., 3-4-1 Nihonbashi-Honcho, Chuo-ku, Tokyo, 103-0023 Japan; 3 Medical Affairs Department, Torii Pharmaceutical Co., Ltd, 3-4-1 Nihonbashi-Honcho, Chuo-ku, Tokyo, 103-0023 Japan; 4 Pharmacovigilance Department, Torii Pharmaceutical Co., Ltd, 3-4-1 Nihonbashi-Honcho, Chuo-ku, Tokyo, 103-0023 Japan; 5 Harumi Triton Clinic of The Jikei University Hospital Harumi Island Triton Square Office Tower, W3 Floor 1-8-8 Harumi, Chuo-ku, Tokyo, 104-0053 Japan

**Keywords:** Ferric citrate, Chronic kidney disease, Hyperphosphatemia, Dialysis, Non-dialysis-dependent, Long-term safety

## Abstract

**Background:**

Ferric citrate hydrate (FC) is an oral iron-based phosphate binder that is used to treat hyperphosphatemia in patients with chronic kidney disease (CKD). This post-marketing surveillance study was performed to investigate the long-term safety and effectiveness of FC.

**Methods:**

This prospective, multicenter, observational post-marketing surveillance study was performed in a real-world setting in Japan. The study involved CKD patients with hyperphosphatemia receiving FC who were undergoing either hemodialysis or peritoneal dialysis or were non-dialysis-dependent. Adverse drug reactions, iron- and erythrocyte-related parameters (i.e., levels of serum ferritin, transferrin saturation, and hemoglobin), and serum levels of phosphorus, corrected calcium, and intact parathyroid hormone were monitored for up to 104 weeks.

**Results:**

Safety was evaluated in 2723 patients. Of these patients, 20.5% discontinued FC because of adverse events, and 3.9% discontinued FC because of unsatisfactory effectiveness. Iron-related parameters gradually increased after the initiation of FC treatment but stabilized after week 36. Effectiveness was analyzed in 2367 patients. Serum phosphorus immediately decreased, and the effect persisted for 104 weeks.

**Conclusion:**

In this 104 week surveillance study, no new safety concerns were noted. The safety profile was not obviously different from those in pre-approval clinical trials and the 52 week interim report of this surveillance study. The serum ferritin level of most patients was below the upper limit of the target range, and iron overload risk was not evident. Long-term FC treatment effectively controlled serum phosphorus.

## Introduction

In patients with chronic kidney disease (CKD), declining kidney function results in CKD-mineral and bone disorder (CKD-MBD), which includes hyperphosphatemia, hypercalcemia, hyperparathyroidism, and impaired bone metabolism. These disorders lead to cardiovascular calcification and bone fractures, resulting in higher morbidity and mortality in CKD patients than in healthy adults [1, 2], therefore, effective management of CKD-MBD is important.

In CKD patients, a high serum phosphate level is associated with CKD progression [3, 4], cardiovascular calcification [5], and all-cause mortality [6]. The Japanese CKD-MBD guidelines recommend the maintenance of the serum phosphorus (*P*) level within the range of 3.5–6.0 mg/dL in CKD patients undergoing hemodialysis and peritoneal dialysis [7]. Several phosphate binders are currently available in Japan, including calcium carbonate, sevelamer hydrochloride, bixalomer, lanthanum carbonate, sucroferric oxyhydroxide, and ferric citrate hydrate (FC) (Riona^®^; Torii Pharmaceutical Co., Ltd., Tokyo, Japan). FC is an oral iron-based phosphate binder that effectively controls the serum *P* concentration in patients undergoing hemodialysis [8] or peritoneal dialysis [9] and in non-dialysis-dependent CKD patients [10]. In Japan, FC has been approved to treat hyperphosphatemia in patients undergoing dialysis and in non-dialysis-dependent patients since 2014. In the United States and Taiwan, ferric citrate (Auryxia^®^; Akebia Therapeutics, Inc., Cambridge, MA, USA and Panion & BF Biotech Inc., Taipei, Taiwan) has been approved to treat hyperphosphatemia in patients undergoing dialysis. Ferric citrate has also been shown to increase the hemoglobin level in patients with iron-deficiency anemia without CKD [11], with CKD undergoing hemodialysis [12], and with non-dialysis-dependent CKD [13]. In Japan, FC was approved to treat iron-deficiency anemia in March 2021, and ferric citrate has also been approved to treat iron-deficiency anemia in patients with non-dialysis-dependent CKD in the United States. With regard to the effect of FC on increasing hemoglobin, iron overload may be a risk factor for long-term use of FC in patients with CKD, which requires long-term safety monitoring of the serum hemoglobin and ferritin levels.

We herein report the final analysis results from a post-marketing surveillance study of FC in CKD patients with hyperphosphatemia who were undergoing either hemodialysis or peritoneal dialysis or were non-dialysis-dependent in a real-world clinical setting in Japan. The purpose of this surveillance study was to confirm the safety, especially iron-related parameters, and effectiveness of FC in a large number of participants undergoing long-term treatment (up to 2 years) in real-world practice.

## Materials and methods

### Surveillance design

This prospective, multicenter, observational post-marketing surveillance study was conducted in Japan. Patients were centrally registered from January 30, 2015, and the survey was terminated on April 30, 2020. The observation period was a maximum of 2 years after treatment initiation. Case report forms were collected for four periods: from treatment initiation to < 3 months, from ≥ 3 to < 6 months, from ≥ 6 to < 12 months, and at ≥ 12 months (up to a maximum of 2 years).

### Patients

CKD patients who were undergoing hemodialysis and/or peritoneal dialysis or were non-dialysis-dependent were eligible for this surveillance study. Patients were registered within 14 days from the initiation of FC treatment and prospectively followed up for a maximum of 2 years. Those who did not return after the first visit and those who were not registered within 14 days after treatment initiation were excluded from the analysis. All other patients were analyzed for safety (safety analysis set). Among the safety analysis set, patients in whom effectiveness could not be evaluated and those who were found not to have met the inclusion criteria were excluded; the remaining patients were analyzed for effectiveness (effectiveness analysis set).

### FC treatment

FC (250 mg tablets containing approximately 60 mg of elemental ferric iron) was to be taken orally three times per day immediately after a meal at a starting dose of 500 mg (1500 mg/day) as recommended in the Japanese package insert. The dose was adjusted according to the serum *P* concentration or clinical status. The maximum dosage allowed was 6000 mg/day. Concomitant medications, such as other phosphate binders or iron preparations, were allowed.

### Safety evaluation

Safety was analyzed in the safety analysis set, and the patients were divided into four groups according to the dialysis methods and status at registration: hemodialysis (HD) group, peritoneal dialysis (PD) group, non-dialysis-dependent (ND) group, and other treatment (OT) group (i.e., combination of hemodialysis and peritoneal dialysis).

All adverse drug reactions (ADRs) were recorded for each group. Serious ADRs were defined as any ADRs that may potentially cause disabilities, hospitalization, or death. All ADRs were categorized using the preferred terms defined in MedDRA version 23.0. In this report, the frequency of hyperferritinemia was coded as a serum ferritin increase because there was no predetermined criterion to distinguish between hyperferritinemia and a serum ferritin increase. The frequencies of polycythemia, red blood cell count increase, and hematocrit increase were coded as a hemoglobin increase.

The levels of iron- and erythrocyte-related parameters, including serum ferritin, transferrin saturation (TSAT), and hemoglobin, were measured as parameters of special interest at each site using its routine measurement methods at the initiation of FC treatment; at 4, 12, 16, 24, 28, 36, 52, 76, and 104 weeks after the initiation of FC treatment; and at discontinuation of FC treatment. The results were summarized for the HD, PD, and ND groups. The OT group was excluded from this analysis because of its small size.

### Effectiveness evaluation

The serum *P*, corrected calcium (cCa), and intact parathyroid hormone (iPTH) levels were monitored to evaluate treatment effectiveness in the effectiveness analysis set. These parameters were measured at each site using its routine measurement methods at the initiation of FC treatment; at 4, 12, 16, 24, 28, 36, 52, 76, and 104 weeks after the initiation of FC treatment; and at treatment discontinuation. The cCa was calculated using absolute values of serum calcium [mg/dL] and serum albumin [g/dL] as 1) when serum albumin was < 4.0 g/dL, cCa [mg/dL] = (absolute value of serum calcium) + [4–(serum albumin)]; and 2) when serum albumin was ≥ 4.0 g/dL, cCa [mg/dL] = absolute value of serum calcium. The values were summarized in the HD, PD, and ND groups. The OT group was excluded from this analysis because of its small size. Furthermore, nine-section charts were made based on the levels of serum *P* and cCa in HD and PD patients to evaluate treatment effectiveness. This chart was not used for ND patients, because it is only recommended for patients receiving dialysis for clinical decision-making in the treatment of hypercalcemia and hyperphosphatemia in Japan [7].

### Statistics

Demographics and ADR frequency among the patients in the safety analysis set were descriptively summarized for each treatment group.

Based on the data from all Japanese pre-approval clinical studies, we planned to enroll 1000 patients in the HD group, 100 in the PD group, and 500 in the ND group. This would allow the identification of an evaluable number of ADRs (i.e., serum ferritin increase and hemoglobin increase) in each group during the 104 week follow-up period.

## Results

### Patient demographics and treatments

**Fig. 1:**
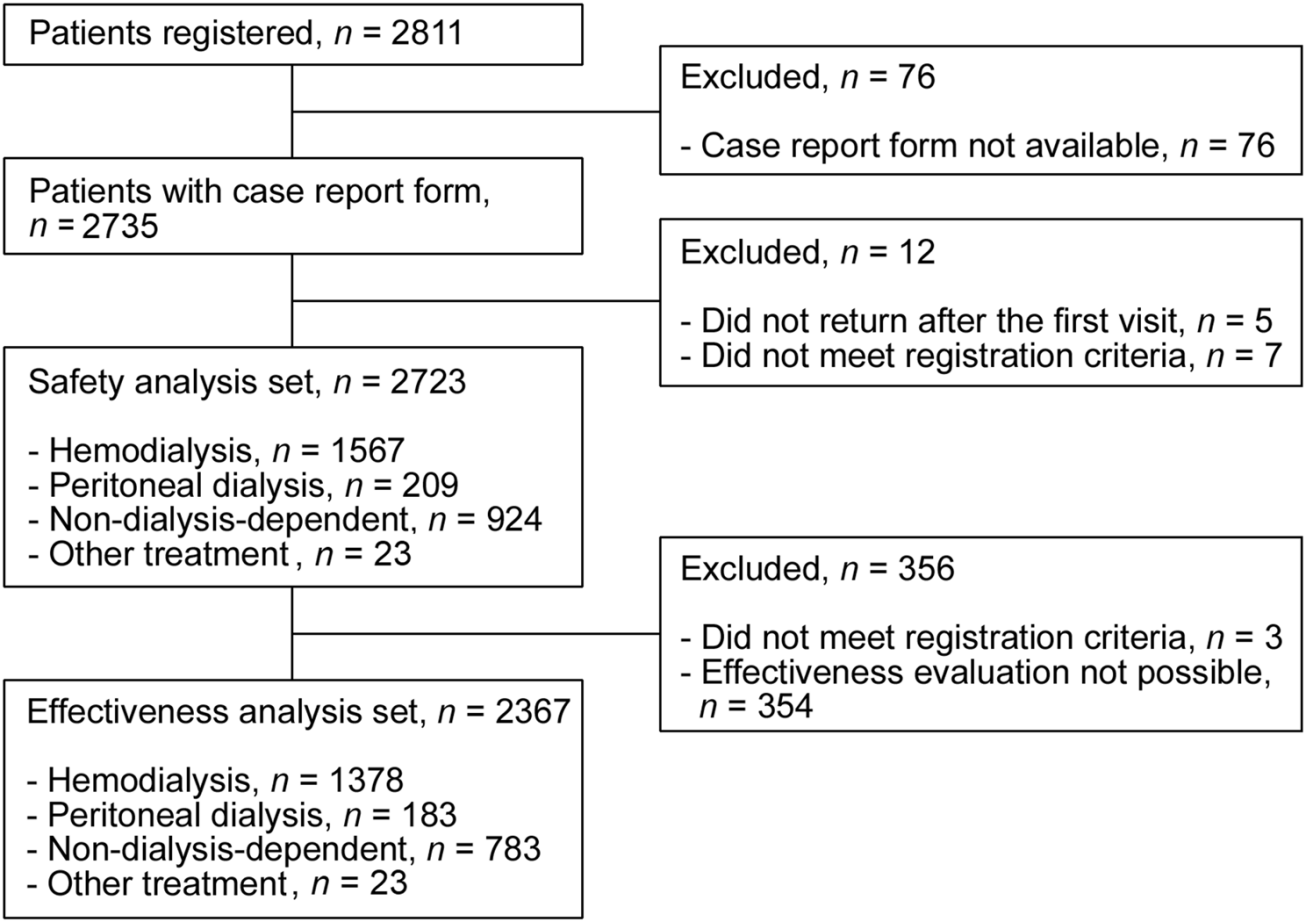
Patient flow

The patients’ demographics and treatments were summarized in the safety analysis set of 2723 patients, including 1567 in the HD, 209 in the PD, 924 in the ND, and 23 in the OT groups (Table 1). During the surveillance period, 2811 patients from 573 facilities were registered, and 2735 case report forms from 558 facilities were collected (Fig. 1). The mean age ± standard deviation (SD) was 65.6 ± 13.0 years and the proportion of men was 60.34% in the overall population, and there was no notable difference among patients in the HD, PD, ND, and OT groups. The most frequent primary disease in the overall population was diabetic kidney disease (41.0%), followed by chronic glomerulonephritis, including IgA nephropathy (23.4%) and nephrosclerosis (18.0%).

**Table 1:** Patient demographics and treatments in each group (safety analysis set)

	Total *n *(%)	HD *n *(%)	PD *n* (%)	ND *n* (%)	OT *n* (%)
Safety analysis set	2723 (100.00)	1567 (100.00)	209 (100.00)	924 (100.00)	23 (100.00)
Sex					
Male	1643 (60.34)	992 (63.31)	142 (67.94)	493 (53.35)	16 (69.57)
Female	1080 (39.66)	575 (36.69)	67 (32.06)	431 (46.65)	7 (30.43)
Age [years]					
< 20	0 (0.00)	0 (0.00)	0 (0.00)	0 (0.00)	0 (0.00)
≥ 20 to < 30	14 (0.51)	7 (0.45)	0 (0.00)	7 (0.76)	0 (0.00)
≥ 30 to < 40	72 (2.64)	39 (2.49)	8 (3.83)	24 (2.60)	1 (4.35)
≥ 40 to < 50	258 (9.47)	145 (9.25)	22 (10.53)	87 (9.42)	4 (17.39)
≥ 50 to < 60	466 (17.11)	255 (16.27)	46 (22.01)	159 (17.21)	6 (26.09)
≥ 60 to < 70	817 (30.00)	504 (32.16)	65 (31.10)	243 (26.30)	5 (21.74)
≥ 70 to < 80	693 (25.45)	408 (26.04)	46 (22.01)	232 (25.11)	7 (30.43)
≥ 80	403 (14.80)	209 (13.34)	22 (10.53)	172 (18.61)	0 (0.00)
Mean ± standard deviation	65.6 ± 13.0	65.6 ± 12.7	63.4 ± 12.7	66.2 ± 13.6	61.1 ± 11.5
Study visit					
Hospitalization	126 (4.63)	39 (2.49)	20 (9.57)	65 (7.03)	2 (8.70)
Ambulatory	2597 (95.37)	1528 (97.51)	189 (90.43)	859 (92.97)	21 (91.30)
Primary disease underlying CKD^a^					
Diabetic kidney disease	1116 (40.98)	650 (41.48)	80 (38.28)	377 (40.80)	9 (39.13)
Chronic glomerulonephritis, including IgA nephropathy	638 (23.43)	393 (25.08)	59 (28.23)	178 (19.26)	8 (34.78)
Nephrosclerosis	490 (17.99)	243 (15.51)	48 (22.97)	197 (21.32)	2 (8.70)
Polycystic kidney disease	125 (4.59)	64 (4.08)	6 (2.87)	55 (5.95)	0 (0.00)
Others	179 (6.57)	100 (6.38)	6 (2.87)	70 (7.58)	3 (13.04)
Unknown	284 (10.43)	168 (10.72)	15 (7.18)	99 (10.71)	2 (8.70)
Dialysis vintage [years]					
< 0.5	212 (7.79)	167 (10.66)	45 (21.53)	0 (0.00)	0 (0.00)
≥ 0.5 to < 1	227 (8.34)	192 (12.25)	32 (15.31)	0 (0.00)	3 (13.04)
≥ 1 to < 3	379 (13.92)	306 (19.53)	67 (32.06)	0 (0.00)	6 (26.09)
≥ 3 to < 5	260 (9.55)	214 (13.66)	40 (19.14)	0 (0.00)	6 (26.09)
≥ 5 to < 10	385 (14.14)	356 (22.72)	24 (11.48)	0 (0.00)	5 (21.74)
≥ 10 to < 20	246 (9.03)	242 (15.44)	1 (0.48)	0 (0.00)	3 (13.04)
≥ 20	89 (3.27)	89 (5.68)	0 (0.00)	0 (0.00)	0 (0.00)
Unknown	925 (33.97)	1 (0.06)	0 (0.00)	924 (100.00)	0 (0.00)
Complications					
Absent	96 (3.53)	76 (4.85)	3 (1.44)	16 (1.73)	1 (4.35)
Present^a^	2623 (96.33)	1488 (94.96)	206 (98.56)	908 (98.27)	21 (91.30)
Gastrointestinal disorders	1214 (44.58)	831 (53.03)	93 (44.50)	281 (30.41)	9 (39.13)
Cardiovascular disorders	2363 (86.78)	1299 (82.90)	192 (91.87)	852 (92.21)	20 (86.96)
Liver disorders	174 (6.39)	101 (6.45)	14 (6.70)	59 (6.39)	0 (0.00)
Metabolic disorders	2132 (78.30)	1110 (70.84)	179 (85.65)	825 (89.29)	18 (78.26)
Others	2278 (83.66)	1336 (85.26)	182 (87.08)	740 (80.09)	20 (86.96)
Unknown	4 (0.15)	3 (0.19)	0 (0.00)	0 (0.00)	1 (4.35)
Any concomitant drugs					
No	13 (0.48)	4 (0.26)	0 (0.00)	9 (0.97)	0 (0.00)
Yes	2710 (99.52)	1563 (99.74)	209 (100.00)	915 (99.03)	23 (100.00)
Phosphate binders^a^					
No	1301 (47.78)	537 (34.27)	82 (39.23)	679 (73.48)	3 (13.04)
Yes	1422 (52.22)	1030 (65.73)	127 (60.77)	245 (26.52)	20 (86.96)
Precipitated calcium carbonate	971 (35.66)	689 (43.97)	75 (35.89)	195 (21.10)	12 (52.17)
Sevelamer hydrochloride	173 (6.35)	157 (10.02)	9 (4.31)	3 (0.32)	4 (17.39)
Bixalomer	117 (4.30)	88 (5.62)	17 (8.13)	11 (1.19)	1 (4.35)
Lanthanum carbonate hydrate	601 (22.07)	474 (30.25)	57 (27.27)	61 (6.60)	9 (39.13)
Sucroferric oxyhydroxide	34 (1.25)	28 (1.79)	2 (0.96)	4 (0.43)	0 (0.00)
Dried aluminum hydroxide gel, magnesium hydroxide	1 (0.04)	1 (0.06)	0 (0.00)	0 (0.00)	0 (0.00)
Secondary hyperparathyroidism drugs					
No	838 (30.77)	297 (18.95)	41 (19.62)	497 (53.79)	3 (13.04)
Yes	1885 (69.23)	1270 (81.05)	168 (80.38)	427 (46.21)	20 (86.96)
Erythropoiesis-stimulating agents					
No	247 (9.07)	117 (7.47)	4 (1.91)	125 (13.53)	1 (4.35)
Yes	2476 (90.93)	1450 (92.53)	205 (98.09)	799 (86.47)	22 (95.65)
Gastric secretion inhibitors					
No	1286 (47.23)	613 (39.12)	104 (49.76)	557 (60.28)	12 (52.17)
Yes	1437 (52.77)	954 (60.88)	105 (50.24)	367 (39.72)	11 (47.83)
Iron preparations					
No	2213 (81.27)	1158 (73.90)	191 (91.39)	844 (91.34)	20 (86.96)
Yes	510 (18.73)	409 (26.10)	18 (8.61)	80 (8.66)	3 (13.04)
Others					
No	239 (8.78)	139 (8.87)	28 (13.40)	69 (7.47)	3 (13.04)
Yes	2484 (91.22)	1428 (91.13)	181 (86.60)	855 (92.53)	20 (86.96)
Average daily dose [mg]					
< 500	174 (6.39)	68 (4.34)	23 (11.00)	81 (8.77)	2 (8.70)
≥ 500 to < 1000	1504 (55.23)	784 (50.03)	105 (50.24)	607 (65.69)	8 (34.78)
≥ 1000 to < 1500	320 (11.75)	205 (13.08)	28 (13.40)	83 (8.98)	4 (17.39)
≥ 1500 to < 2000	628 (23.06)	430 (27.44)	46 (22.01)	144 (15.58)	8 (34.78)
≥ 2000 to < 2500	72 (2.64)	60 (3.83)	5 (2.39)	6 (0.65)	1 (4.35)
≥ 2,500 to < 3000	17 (0.62)	15 (0.96)	1 (0.48)	1 (0.11)	0 (0.00)
≥ 3000	8 (0.29)	5 (0.32)	1 (0.48)	2 (0.22)	0 (0.00)
Mean ± standard deviation	995.1 ± 496.9	1079.6 ± 511.2	972.0 ± 499.4	853.6 ± 436.0	1126.3 ± 485.1
Mean treatment period [days]					
Mean ± standard deviation	419.3 ± 280.1	465.8 ± 281.0	402.2 ± 272.5	340.3 ± 262.8	587.9 ± 188.6

*HD* hemodialysis, *PD* peritoneal dialysis, *ND* non-dialysis-dependent, *OT* other treatment, *CKD* chronic kidney disease, *IgA* immunoglobulin A

^a^Multiple answers possible

Overall, 2710/2723 patients (99.52%) received concomitant medications. About half of the overall population (1422/2723 patients; 52.22%) received hyperphosphatemia therapy other than FC (Table 1). The most commonly used hyperphosphatemia therapy was precipitated calcium carbonate (971/2723 patients, 35.66%), followed by lanthanum carbonate hydrate (601/2723 patients, 22.07%), and sevelamer hydrochloride (173/2723 patients, 6.35%). Gastric secretion inhibitors, such as histamine type-2 receptor antagonists, proton pump inhibitors, and medication to prevent non-steroidal anti-inflammatory drug-induced ulcers, were used in 1437/2723 patients (52.77%). Erythropoiesis-stimulating agents (ESAs) were used in 2476/2723 patients (90.93%), and iron preparations were used in 510/2723 patients (18.73%).

All 2723 patients received FC. In total, 1692/2723 patients (62.1%) discontinued the treatment (Table 2). The reason for discontinuation was adverse events in 558/2723 patients (20.5%) and unsatisfactory effectiveness in 105/2723 patients (3.9%). The mean treatment period ± SD of FC was 419.3 ± 280.1 days in the overall population, 465.8 ± 281.0 days in the HD, 402.2 ± 272.5 days in the PD, 340.3 ± 262.8 days in the ND, and 587.9 ± 188.6 days in the OT groups (Table 1). The mean daily dose ± SD of FC was 995.1 ± 496.9 mg in the overall population (1079.6 ± 511.2 mg in the HD, 972.0 ± 499.4 mg in the PD, 853.6 ± 436.0 mg in the ND, and 1126.3 ± 485.1 mg in the OT groups).

**Table 2:** Summary of discontinuation (safety analysis set)

	Observation period (months)					
	At initiation	< 3	≥ 3 to < 6	≥ 6 to < 12	≥ 12	Total
Safety analysis set, *n*	2723	2723	2348	1916	1502	2723
HD	1567	1567	1376	1168	986	1567
PD	209	209	182	150	110	209
ND	924	924	767	576	386	924
OT	23	23	23	22	20	23
Treatment discontinuation, *n*	0	375	432	414	471	1692
Adverse events	0	185	114	117	142	558
Effectiveness not satisfactory	0	15	28	30	32	105
Hospital switched; treatment visits stopped	0	81	101	102	115	399
Follow-up not possible	0	0	99	68	74	241
Other reasons	0	94	90	97	108	389

*HD* hemodialysis, *PD* peritoneal dialysis, *ND* non-dialysis-dependent, *OT* other treatment

### Safety

The frequently observed ADRs in each treatment group in the safety analysis set are summarized in Table 3. In total, 445 ADRs were reported in 323/1567 patients (20.61%) in the HD, 67 events in 51/209 patients (24.40%) in the PD, 188 events in 151/924 patients (16.34%) in the ND, and 8 events in 7/23 patients (30.43%) in the OT groups. An increased serum ferritin level was the most commonly reported ADR (4.52%). Gastrointestinal ADRs observed in more than 1% of patients in any group were diarrhea [56/1567 patients (3.57%) in the HD, 13/209 patients (6.22%) in the PD, 42/924 patients (4.55%) in the ND, and 1/23 patients (4.35%) in the OT groups], constipation [28/1567 patients (1.79%) in the HD, 3/209 patients (1.44%) in the PD, 14/924 patients (1.52%) in the ND, and 1/23 patients (4.35%) in the OT groups], and nausea [19/1567 patients (1.21%) in the HD, 1/209 patients (0.48%) in the PD, 10/924 patients (1.08%) in the ND, and 0/23 patients in the OT groups].

**Table 3:** Adverse drug reactions observed in five or more patients in safety analysis set of this study (safety analysis set)

	Pre-approval total^a^	Post-marketing surveillance				
		HD	PD	ND	OT	Total
Safety analysis set, *n*	801	1567	209	924	23	2723
Patients with any ADRs, *n*	204	323	51	151	7	532
ADRs, *n*	–	445	67	188	8	708
Proportion of patients with any ADRs, %	25.47	20.61	24.40	16.34	30.43	19.54
ADRs, *n* (%)						
Serum ferritin increased^b^	22 (2.75)	60 (3.83)	19 (9.09)	40 (4.33)	4 (17.39)	123 (4.52)
Diarrhea	55 (6.87)	56 (3.57)	13 (6.22)	42 (4.55)	1 (4.35)	112 (4.11)
Hemoglobin increased^c^	20 (2.50)	50 (3.19)	0 (0.00)	1 (0.11)	0 (0.00)	51 (1.87)
Constipation	26 (3.25)	28 (1.79)	3 (1.44)	14 (1.52)	1 (4.35)	46 (1.69)
Nausea	7 (0.87)	19 (1.21)	1 (0.48)	10 (1.08)	0 (0.00)	30 (1.10)
Hypertension	4 (0.50)	12 (0.77)	2 (0.96)	3 (0.32)	0 (0.00)	17 (0.62)
Abdominal discomfort	20 (2.50)	12 (0.77)	0 (0.00)	3 (0.32)	0 (0.00)	15 (0.55)
Feces discolored	1 (0.12)	12 (0.77)	0 (0.00)	0 (0.00)	0 (0.00)	12 (0.44)
Abdominal distension	10 (1.25)	9 (0.57)	0 (0.00)	3 (0.32)	0 (0.00)	12 (0.44)
Vomiting	6 (0.75)	7 (0.45)	1 (0.48)	0 (0.00)	0 (0.00)	8 (0.29)
Feces soft	29 (3.62)	6 (0.38)	0 (0.00)	2 (0.22)	0 (0.00)	8 (0.29)
Decreased appetite	3 (0.37)	5 (0.32)	1 (0.48)	4 (0.43)	0 (0.00)	10 (0.37)
Pruritus	2 (0.25)	5 (0.32)	0 (0.00)	3 (0.32)	0 (0.00)	8 (0.29)
Abdominal pain	7 (0.87)	4 (0.26)	1 (0.48)	2 (0.22)	0 (0.00)	7 (0.26)
Gastroesophageal reflux disease	0 (0.00)	3 (0.19)	1 (0.48)	1 (0.11)	0 (0.00)	5 (0.18)
Blood iron increased	0 (0.00)	2 (0.13)	1 (0.48)	2 (0.22)	0 (0.00)	5 (0.18)
Hepatic function abnormal	4 (0.50)	1 (0.06)	2 (0.96)	3 (0.32)	0 (0.00)	6 (0.22)
Renal impairment	0 (0.00)	0 (0.00)	0 (0.00)	5 (0.54)	0 (0.00)	5 (0.18)

*ADR* adverse drug reaction, *HD* hemodialysis, *PD* peritoneal dialysis, *ND* non-dialysis-dependent, *OT* other treatment

^a^Data from pre-approval clinical studies conducted in Japan

^b^Including the occurrence of hyperferritinemia

^c^Including the occurrence of polycythemia, red blood cell count increased, and hematocrit increased

In the overall population, 73 serious ADRs were observed in 60/2723 patients (2.20%), including 37 events in 30/1567 patients (1.91%) in the HD, 6 events in 5/209 patients (2.39%) in the PD, 30 events in 25/924 patients (2.71%) in the ND, and 0 events in 0/23 patients in the OT groups (Table 4). Among those, 17 of all 2723 patients (0.62%) died.

**Table 4:** Serious adverse drug reactions (safety analysis set)

	HD	PD	ND	OT	Total
Safety analysis set, *n*	1567	209	924	23	2723
Patients with any serious ADRs, *n*	30	5	25	0	60
Serious ADRs, *n*	37	6	30	0	73
Proportion of patients with any serious ADRs, %	1.91	2.39	2.71	0.00	2.20
ADR, *n* (*n* of deaths)^a^					
Infections and infestations	1 (1)	1	2 (1)		4 (2)
Peritonitis	1 (1)	1			2 (1)
Urinary tract infection			1		1
Renal cyst infection			1 (1)		1 (1)
Neoplasms benign, malignant and unspecified (including cysts and polyps)	2 (1)				2 (1)
Gastric cancer	1				1
Lymphoma	1 (1)				1 (1)
Blood and lymphatic system disorders			2 (1)		2 (1)
Eosinophilia			2 (1)		2 (1)
Metabolism and nutrition disorders	3	1	1		5
Dehydration	1				1
Hyperphosphatemia	1				1
Hypocalcemia			1		1
Hyponatremia		1			1
Decreased appetite	1				1
Nervous system disorders	8 (2)		2 (1)		10 (3)
Cerebellar infarction	1		1		2
Cerebral hemorrhage	1 (1)				1 (1)
Cerebral infarction	3				3
Subarachnoid hemorrhage	1 (1)		1 (1)		2 (2)
Transient ischemic attack	1				1
Ischemic cerebral infarction	1				1
Cardiac disorders	9 (4)		3 (2)		12 (6)
Acute myocardial infarction	3 (2)				3 (2)
Angina pectoris	2 (1)				2 (1)
Arrhythmia			2 (2)		2 (2)
Arteriosclerosis coronary artery	1				1
Cardiac failure	1 (1)				1 (1)
Cardiac failure chronic	1				1
Cardiac failure congestive	2		1		3
Respiratory, thoracic and mediastinal disorders	2 (1)		1		3 (1)
Pneumonia aspiration	2 (1)				2 (1)
Pulmonary edema			1		1
Gastrointestinal disorders	3 (1)	2			5 (1)
Abdominal pain upper		1			1
Diarrhea	1	1			2
Ileus	1 (1)				1 (1)
Pancreatitis	1				1
Vomiting		1			1
Hepatobiliary disorders			1		1
Hepatic function abnormal			1		1
Musculoskeletal and connective tissue disorders	1				1
Back pain	1				1
Renal and urinary disorders			8 (1)		8 (1)
Renal failure			2		2
Renal impairment			4		4
Acute kidney injury			1 (1)		1 (1)
End stage renal disease			1		1
General disorders and administration site conditions	2 (1)	1 (1)	3 (1)		6 (3)
Chest pain			1		1
Death	1 (1)	1 (1)	1 (1)		3 (3)
Malaise	1				1
Edema due to renal disease			1		1
Investigations	1		6		7
Blood creatinine increased			1		1
Glomerular filtration rate decreased			1		1
Hemoglobin decreased	1				1
Serum ferritin increased			4		4
Injury, poisoning and procedural complications	4 (1)		1		5 (1)
Femoral neck fracture	1 (1)				1 (1)
Shunt occlusion	2		1		3
Pelvic fracture	1				1

*ADR* adverse drug reaction, *HD* hemodialysis, *PD* peritoneal dialysis, *ND* non-dialysis-dependent, OT other treatment

^a^Multiple events in a single patient are possible

### Safety: parameters of special interest

**Fig. 2:**
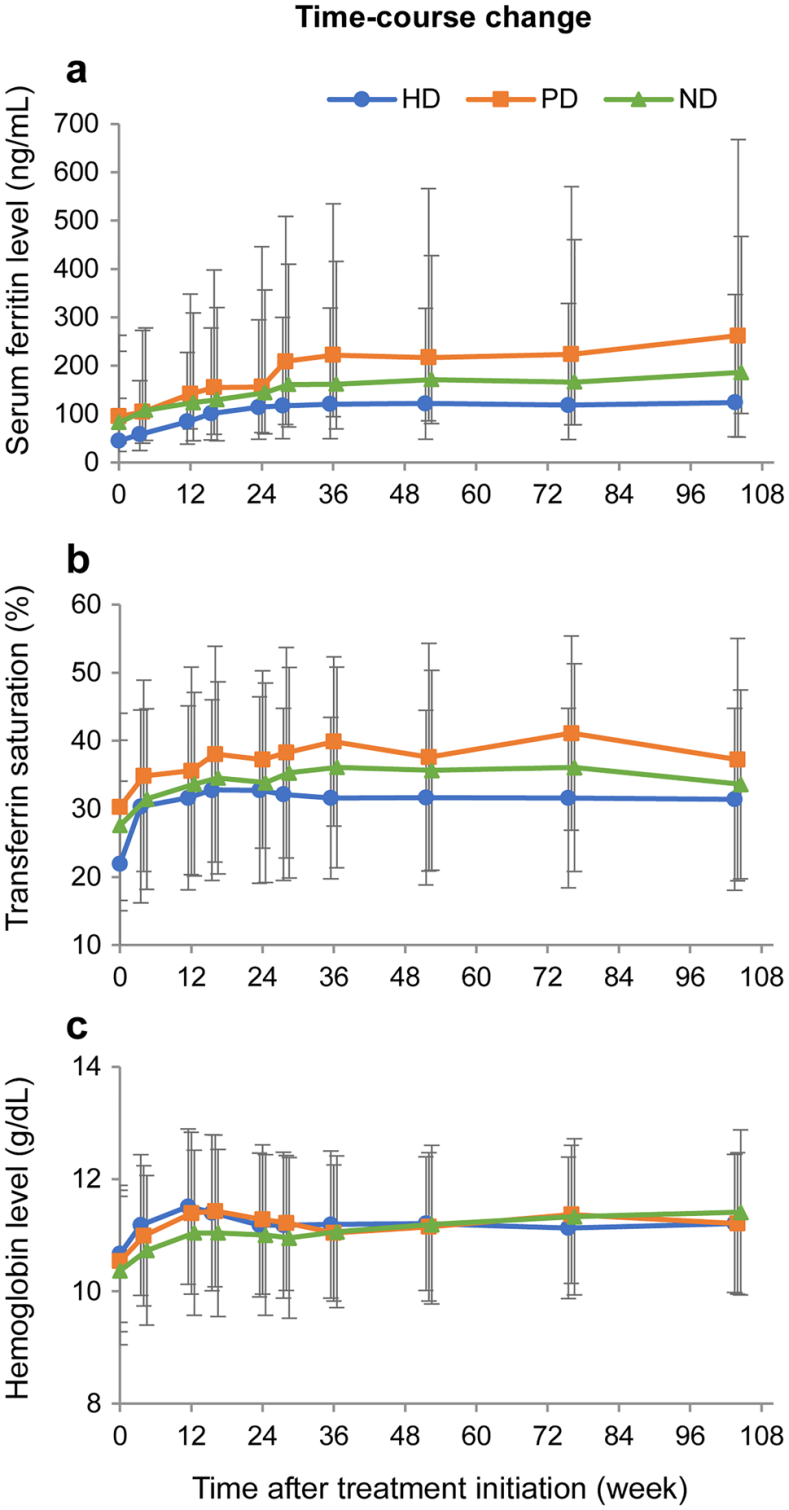
Iron- and erythrocyte-related parameters of special interest (safety analysis set) Time-course changes in **a** serum ferritin, **b** transferrin saturation, and **c** hemoglobin. Data represent **a** median and (**b**, **c**) mean ± standard deviation. Error bars in **a** are from the first to third quartiles. Blue: hemodialysis group (HD), orange: peritoneal dialysis group (PD), green: non-dialysis-dependent group (ND)

A serum ferritin increase, including hyperferritinemia, was observed in 60/1567 patients in the HD (3.83%), 19/209 patients (9.09%) in the PD, 40/924 patients (4.33%) in the ND, and 4/23 patients (17.39%) in the OT groups (Table 3). At treatment initiation, the median (first quartile, third quartile) ferritin levels were 44.50 (21.40, 88.10) ng/mL in the HD, 95.15 (51.00, 167.80) ng/mL in the PD, and 82.65 (43.00, 147.20) ng/mL in the ND groups (Fig. 2a). The levels showed a stable increasing trend, and at week 36 they were 120.70 (72.00, 198.30) ng/mL in the HD, 222.10 (127.80, 312.80) ng/mL in the PD, and 162.00 (93.00, 254.00) ng/mL in the ND groups. The levels subsequently showed the tendency to stabilize: the ferritin levels at week 104 were 124.00 (71.00, 223.00) ng/mL in the HD, 262.10 (209.55, 405.60) ng/mL in the PD, and 186.00 (84.90, 281.50) ng/mL in the ND groups. The time-course changes in the TSAT levels were similar to those of the serum ferritin levels. At FC treatment initiation, the mean ± SD TSAT was 21.97 ± 12.09% in the HD, 30.30 ± 13.77% in the PD, and 27.58 ± 12.52% in the ND groups (Fig. 2b). The levels showed a gradually increasing trend, and at week 36 they were 31.59 ± 11.83% in the HD, 39.88 ± 12.43% in the PD, and 36.11 ± 14.75% in the ND groups. They subsequently showed the tendency to stabilize. The TSAT levels at week 104 were 31.41 ± 13.35% in the HD, 37.24 ± 17.78% in the PD, and 33.61 ± 13.85% in the ND groups. The mean hemoglobin level showed an increasing trend in all treatment groups. The mean ± SD hemoglobin levels at treatment initiation were 10.67 ± 1.22 g/dL in the HD, 10.54 ± 1.26 g/dL in the PD, and 10.37 ± 1.32 g/dL in the ND groups (Fig. 2c). The levels at week 104 were 11.21 ± 1.23 g/dL in the HD, 11.21 ± 1.26 g/dL in the PD, and 11.41 ± 1.47 g/dL in the ND groups. A serious serum ferritin increase was observed in 4/2723 patients (0.15%) overall, all of whom were in the ND group (4/924, 0.43%) (Table 4). The physicians subjectively determined the seriousness of the serum ferritin increase because of the lack of predetermined criteria. Although iron overload is associated with an increased risk of hepatic dysfunction or infectious disease, such ADRs were not observed in these four patients with a serious serum ferritin increase. No other serious ADRs involving parameters of special interest were observed.

### Effectiveness

**Fig. 3:**
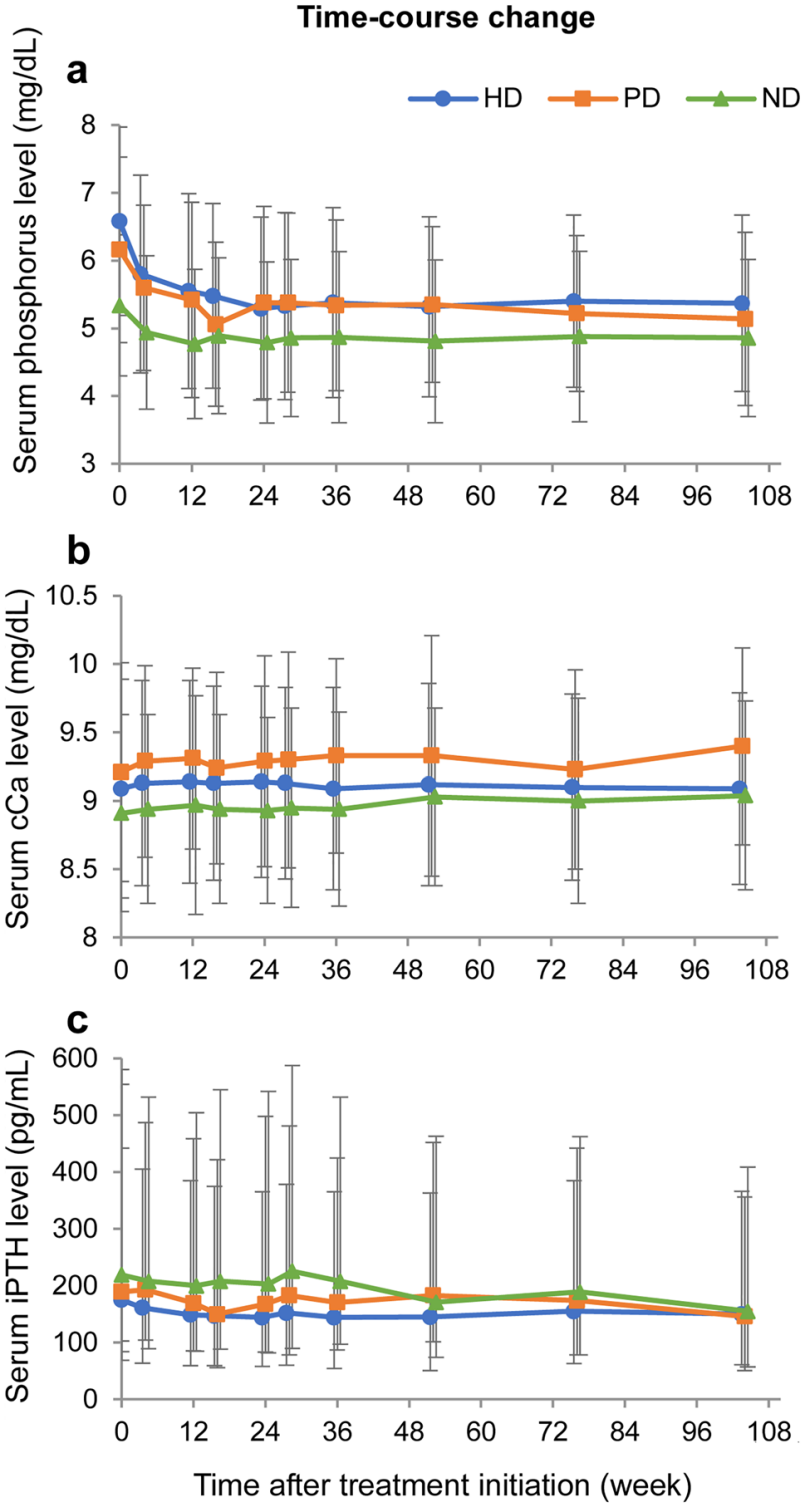
Effectiveness evaluation parameters (effectiveness analysis set) Time-course changes in mean **a** serum phosphorus, **b** corrected calcium (cCa), and **c** intact parathyroid hormone (iPTH). Data represent (**a**, **b**) mean ± standard deviation and **c** median. Error bars in **c** are from the first to third quartiles. Blue: hemodialysis group (HD), orange: peritoneal dialysis group (PD), green: non-dialysis-dependent group (ND)

**Fig. 4:**
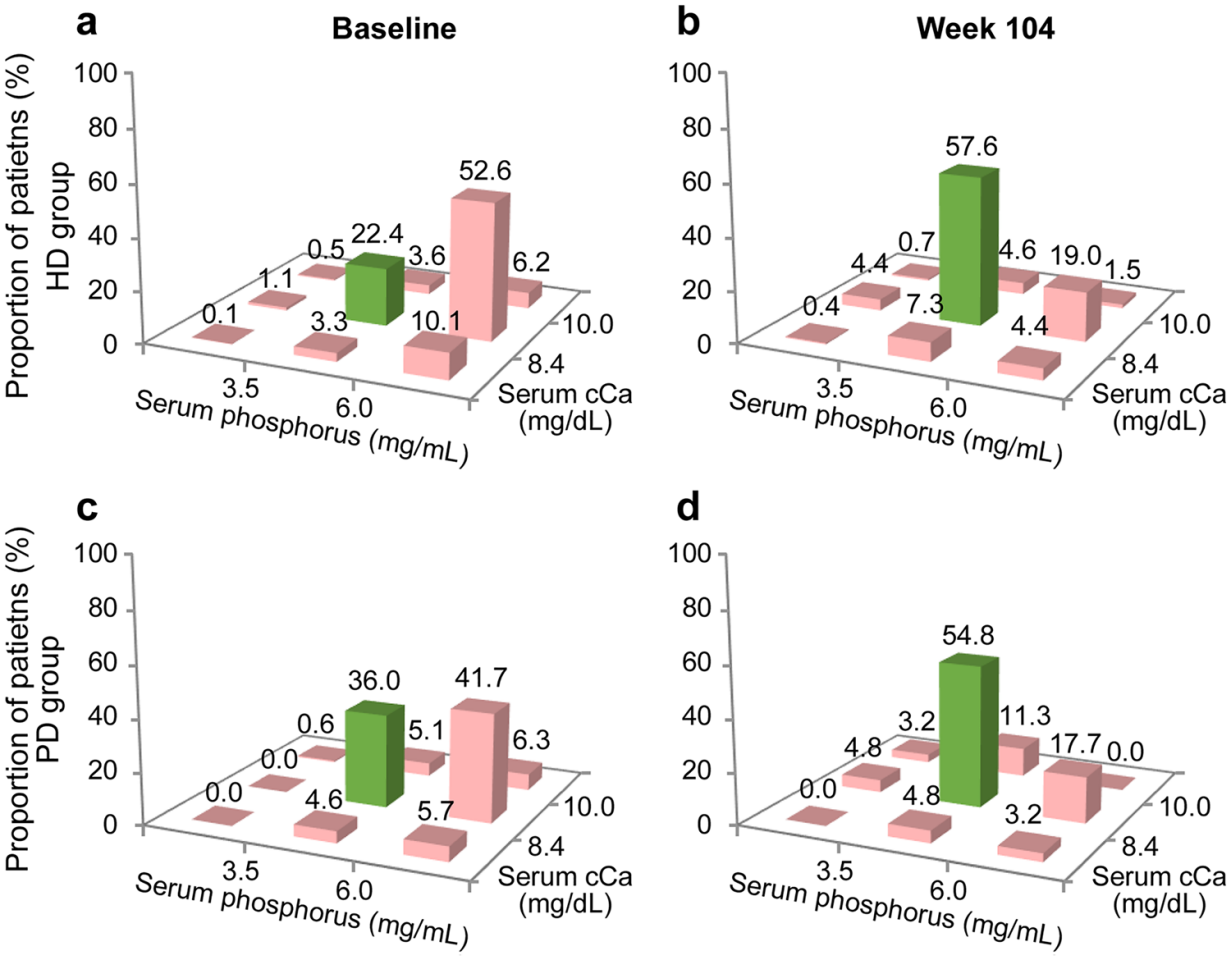
Nine-section charts (effectiveness analysis set) Nine-section charts for patients undergoing (**a**, **b**) hemodialysis (HD) and (**c**, **d**) peritoneal dialysis (PD) at (**a**, **c**) baseline and (**b**, **d**) week 104. Green bars designate well-managed serum phosphorus and corrected calcium (cCa)

The effectiveness analysis set comprised 2367 patients (1378 in the HD, 183 in the PD, 783 in the ND, and 23 in the OT groups). After the FC treatment initiation, the serum *P* level showed an immediate decreasing trend in all groups (Fig. 3a). At FC treatment initiation, the mean serum *P* level ± SD was 6.58 ± 1.39 mg/dL in the HD, 6.16 ± 1.37 mg/dL in the PD, and 5.34 ± 1.04 mg/dL in the ND groups. At week 104, the mean serum *P* level ± SD was 5.37 ± 1.30 mg/dL in the HD, 5.14 ± 1.28 mg/dL in the PD, and 4.86 ± 1.16 mg/dL in the ND groups. The levels of serum cCa (Fig. 3b) and iPTH (Fig. 3c) did not substantially change in any group. Figure 4 shows the nine-section charts for HD and PD patients at baseline and at week 104. The center section shows that the target ranges of both serum *P* (3.5–6.0 mg/dL) and serum cCa (8.4–10.0 mg/dL) were achieved [7]. At baseline, proportions of patients in the center section were 22.4% in the HD and 36.0% in the PD groups (Fig. 4a, c), indicating insufficient serum *P* and cCa management. At 104 weeks after FC treatment initiation, the highest proportion of patients was in the center section (57.6% in the HD and 54.8% in the PD groups) (Fig. 4b, d).

## Discussion

The ferric iron from FC binds to ingested *P* in the gastrointestinal tract and forms insoluble ferric phosphate, which promotes fecal *P* excretion [14]. Pre-approval clinical trials for CKD patients with hyperphosphatemia showed that FC is associated with gastrointestinal ADRs: Among all Japanese pre-approval clinical trials, 55/801 patients (6.87%) had diarrhea and 26/801 patients (3.25%) had constipation (Table 3). Neither of these ADRs was more frequent after the long-term use of FC in the present surveillance. All ADRs have been previously described in pre-approval clinical studies or the 52 week interim surveillance study. There were no obvious differences in ADR frequencies among the HD, PD, ND, and OT groups. Overall, the ADR profile in this 104 week surveillance study was similar to that in pre-approval clinical trials and the interim results of the current surveillance study [15].

Iron- and erythrocyte-related parameters were evaluated as parameters of special interest because FC is an iron-based phosphate binder; therefore, FC may induce iron deposition in organs [16] and increase the risk of diseases associated with iron overload, especially hepatic dysfunction.

The Japanese Society for Dialysis Therapy (JSDT) guidelines for renal anemia in patients with CKD recommend that the serum ferritin level should not exceed 300 ng/mL [17]. The serum ferritin level and TSAT level in the present surveillance study showed a gradually increasing trend after treatment initiation, and after week 36 they stayed at levels similar to those in the interim surveillance study [15]. The PD group showed the highest serum ferritin level, which was in line with previously reported clinical studies [8–10]. In these PD patients, median ferritin levels did not exceed 300 ng/mL (262.1 ng/mL) and the hemoglobin level was well maintained within the target range recommended in the JSDT guidelines [17]. The importance of treating anemia and iron deficiency in CKD patients has recently been receiving increasing attention, as several studies have demonstrated that these conditions are associated with an increased risk of hospitalizations, cardiovascular events, or all-cause mortality [18, 19]. FC is expected to contribute to the treatment of anemia and iron deficiency in CKD patients, but caution regarding iron overload is required, especially in PD patients. Therefore, continuous monitoring is warranted to prevent iron overload and excessive hematopoiesis in CKD patients treated with FC, and adjusting the dose of FC should be considered accordingly. It may be possible to prevent iron overload by adjusting the dose of concomitant medications, such as ESAs or iron preparations. In this study, the data were not available to evaluate if FC changed the required dose of ESAs or iron preparations. Further data accumulation is therefore necessary.

Administration of FC with other concomitant drugs effectively managed the serum *P* level in CKD patients undergoing hemodialysis and peritoneal dialysis and in patients who were non-dialysis-dependent. Additionally, the effect was present after long-term treatment without largely affecting the levels of cCa or iPTH. In the nine-section charts, the proportions of patients within the center section were increased by FC treatment in both the HD and PD groups, and were not decreased after the long-term treatment. These results demonstrate that FC with other concomitant therapy successfully managed CKD-MBD.

Limitations of this surveillance study include the many missing data because of the observational nature of the surveillance, the lack of a control group for comparison, and possible bias in patient selection because only patients with favorable safety and effectiveness outcomes continued FC treatment for the long term.

## Conclusions

In this prospective, multicenter, observational post-marketing surveillance study of the treatment of hyperphosphatemia with FC, no new safety issues emerged during the 104 week observation period. A risk of iron overload was not evident; the serum ferritin level of most patients remained at < 300 ng/mL, the recommended upper limit of the target range in Japan. Additionally, no signs of hepatic dysfunction were observed in patients with hyperferritinemia. However, further observation is needed. FC effectively controlled the serum *P* concentration in CKD patients with hyperphosphatemia who were undergoing either hemodialysis or peritoneal dialysis or were non-dialysis-dependent.

## Data Availability

The datasets generated and/or analyzed during the study are available from the corresponding author on reasonable request.
